# Introducing TreeCollapse: a novel greedy algorithm to solve the cophylogeny reconstruction problem

**DOI:** 10.1186/1471-2105-15-S16-S14

**Published:** 2014-12-08

**Authors:** Benjamin Drinkwater, Michael A Charleston

**Affiliations:** 1 School of Information Technologies, 1 Cleveland St, 2006 University of Sydney, Australia Full list of author information is available at the end of the article

**Keywords:** Coevolution, Phylogenetics, Cophylogeny, Greedy Algorithms, NP-Hard

## Abstract

**Background:**

Cophylogeny mapping is used to uncover deep coevolutionary associations between two or more phylogenetic histories at a macro coevolutionary scale. As cophylogeny mapping is NP-Hard, this technique relies heavily on heuristics to solve all but the most trivial cases. One notable approach utilises a metaheuristic to search only a subset of the exponential number of fixed node orderings possible for the phylogenetic histories in question. This is of particular interest as it is the only known heuristic that guarantees biologically feasible solutions. This has enabled research to focus on larger coevolutionary systems, such as coevolutionary associations between figs and their pollinator wasps, including over 200 taxa. Although able to converge on solutions for problem instances of this size, a reduction from the current cubic running time is required to handle larger systems, such as Wolbachia and their insect hosts.

**Results:**

Rather than solving this underlying problem optimally this work presents a greedy algorithm called TreeCollapse, which uses common topological patterns to recover an approximation of the coevolutionary history where the internal node ordering is fixed. This approach offers a significant speed-up compared to previous methods, running in linear time. This algorithm has been applied to over 100 well-known coevolutionary systems converging on Pareto optimal solutions in over 68% of test cases, even where in some cases the Pareto optimal solution has not previously been recoverable. Further, while TreeCollapse applies a local search technique, it can guarantee solutions are biologically feasible, making this the fastest method that can provide such a guarantee.

**Conclusion:**

As a result, we argue that the newly proposed algorithm is a valuable addition to the field of coevolutionary research. Not only does it offer a significantly faster method to estimate the cost of cophylogeny mappings but by using this approach, in conjunction with existing heuristics, it can assist in recovering a larger subset of the Pareto front than has previously been possible.

## Background

Ecologically linked groups of organisms often place selective pressures on one another driving the evolutionary process [1]. These selective pressures give rise to tightly coupled coevolutionary systems. Coevolution occurs in a wide variety of biological systems including host-parasite relationships [2], mimicry between species [3], biogeography [4], insect-plant interactions [5], host-pathogen networks [6], and predator-prey systems [7].

The study of the macro scale coevolutionary associations (*φ*) formed between the host and parasite phylogenies is encompassed by the field of cophylogenetics [8]. Analysis of these systems can be applied to tackle some of the most pressing global health issues facing society today [9]. Coevolutionary analysis of systems such as primates and malaria-causing *Plasmodium *[6] offer the potential to provide further insights into this deadly disease. Although encompassing a large number of biological scenarios each coevolutionary system consists of an independent evolutionary history often refered to as the *host *phylogeny and its corresponding dependent evolutionary history known as the *parasite *phylogeny [10].

**Figure 1:**
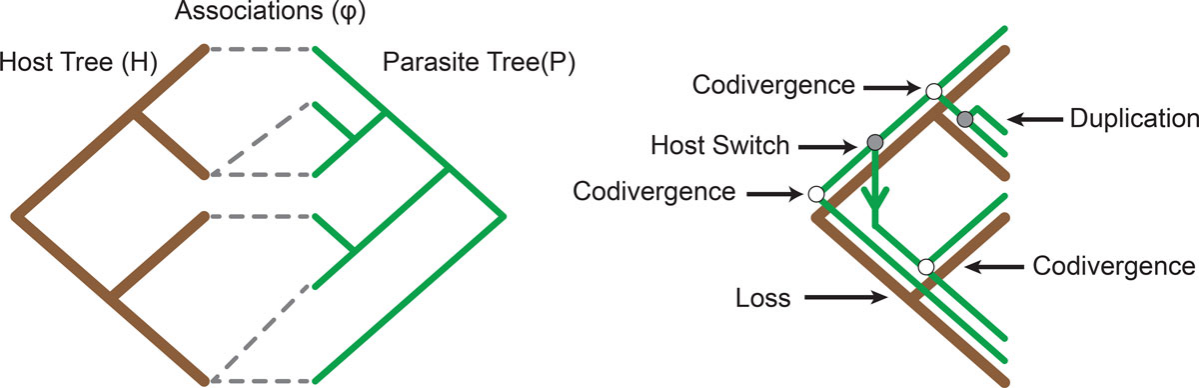
**Tanglegram and Cophylogeny Mapping**. A simple tanglegram where the optimal mapping contains all four recoverable events.

Cophylogenetics aims to provide such insights by evaluating the significance of the observed associations (*φ*) between the host (*H*) and parasite (*P*) phylogenies and the branching patterns between *H *and *P *to identify if these ecologically linked organisms have formed deep coevolutionary bonds or if their evolutionary histories are independent [10]. The host and parasite phylogenies considered herein are bifurcating trees where the total number of nodes for both trees is bound by *O*(*n*). Therefore, cophylogenetics aims to analyse and understand the tuple (*H, P, φ*), often visualised as a tanglegram [11] as seen in Figure 1. As a result, the complexity of this problem is bound in terms of *n*, the size of *H *and *P *.

A common method for evaluating the tuple (*H, P, φ*) is *cophylogeny mapping *where the parasite tree is mapped into the host tree such that the associations (*φ*) are conserved. A cophylogeny mapping (Φ(*P*)) estimates the parasite's evolutionary history with respect to its host using the four recoverable coevolutionary events of *codivergence, duplication, host switch *and *loss*.

A *Codivergence *event is a concurrent divergence of both a host and parasite species [8]. A *Duplication *event, by contrast, is an independent divergence of a parasite species. Following this divergence event both new parasite species continue to track their initial host [10]. A *Host Switch*, similar to a duplication event, is an independent divergence of a parasite species. In contrast to a duplication event, however, one of the new parasite species switches to a new host while the remaining parasite species continues to inhabit the initial host [12]. A *Loss *event represents one of three possibilities: failure to diverge by the parasite species following a divergence event by its host, an extinction event of a parasite species or sampling error in the phylogenetic reconstruction of the parasite phylogeny [13]. These four events when applied together are capable of recovering the full set of mappings possible where each parasite may only inhabit a single host. This is the permutation of the cophylogeny reconstruction problem which is considered herein. An example of all four recoverable events applied to solve a simple tanglegram can be seen in Figure 1. The aim when recovering a cophylogeny mapping is to retrieve a solution where the resultant cost is minimised. The set of all mappings with a minimum cost are contained within the Pareto set [14]. The cophylogeny reconstruction problem is defined as the problem of reconstructing a map from the Pareto set where the mapping cost is minimised, which has been established as NP-Hard [14,15], which has in turn given rise to a number of heuristic approaches. Currently there are two classes of heuristics, pattern and event-based methods. Event-based algorithms have been shown to be the only approach that can guarantee recovered solutions are optimal [8]. Ronquist [16], however, has argued that pattern-based algorithms can produce robust approximations that can be as good as event-based methods which impose constraints upon the solution space. Imposing constraints is required due to the computational intractability of this problem, and therefore pattern-based methods have the potential to offer comparable precision to event-based methods. An existing pattern-based approach is Page's Reconciled Tree Analysis [17], which solves this problem optimally with the condition that host switches are not permitted. This algorithm recovers a Pareto optimal solution in *O*(*n*). This approach, while recovering an optimal solution, is unable to recover solutions which accurately represent coevolutionary interactions where host switch events are prevalent, such as host-pathogen systems or where closely related parasites infect distantly related hosts. Another well known pattern-based method is Brooks Parsimony Analysis (BPA) [18,19]. This method uses a binary coding representation of the parasite phylogeny and uses this binary coding to map the parasite into the host. This method has been shown to produce good solutions [20] but has not yet been implemented due to inconsistencies in the selection of events and the ordering of nodes in the host phylogeny.

Due to the limitations faced by both these approaches, pattern-based algorithms are not often applied to this problem, with the majority of recent cophylogenetic analyses using event-based methods [21-25]. Approximating this problem using event-based methods aims to recover cophylogeny mappings where the global score is minimised. This approach is based on Ronquist's generalised definition of the cophylogeny reconstruction problem, which states that only by minimising the total cost of all events can an algorithm recover a Pareto optimal reconstruction [26]. Two recent algorithmic approaches that have leveraged this generalised definition apply dynamic programming to modified instances of the cophylogeny reconstruction problem. The first approach ignores the relative ordering of the internal nodes of the host tree and the second approach fixes the relative order of the internal nodes of the host tree.

By ignoring the relative order of the internal nodes of the host tree the cophylogeny reconstruction problem can be solved in polynomial time. This approach is derived from a heuristic used to approximate the Feedback Arcs Set Problem which is also NP-Hard [27,28]. Tarzan [27] and CoRe-PA [29] apply this technique to recover cophylogeny mappings in polynomial time. This approach is currently the fastest known approach for recovering solutions where all four recoverable events are permitted with the fastest proposed algorithm running in *O*(*n*^2^) [30].

**Figure 2:**
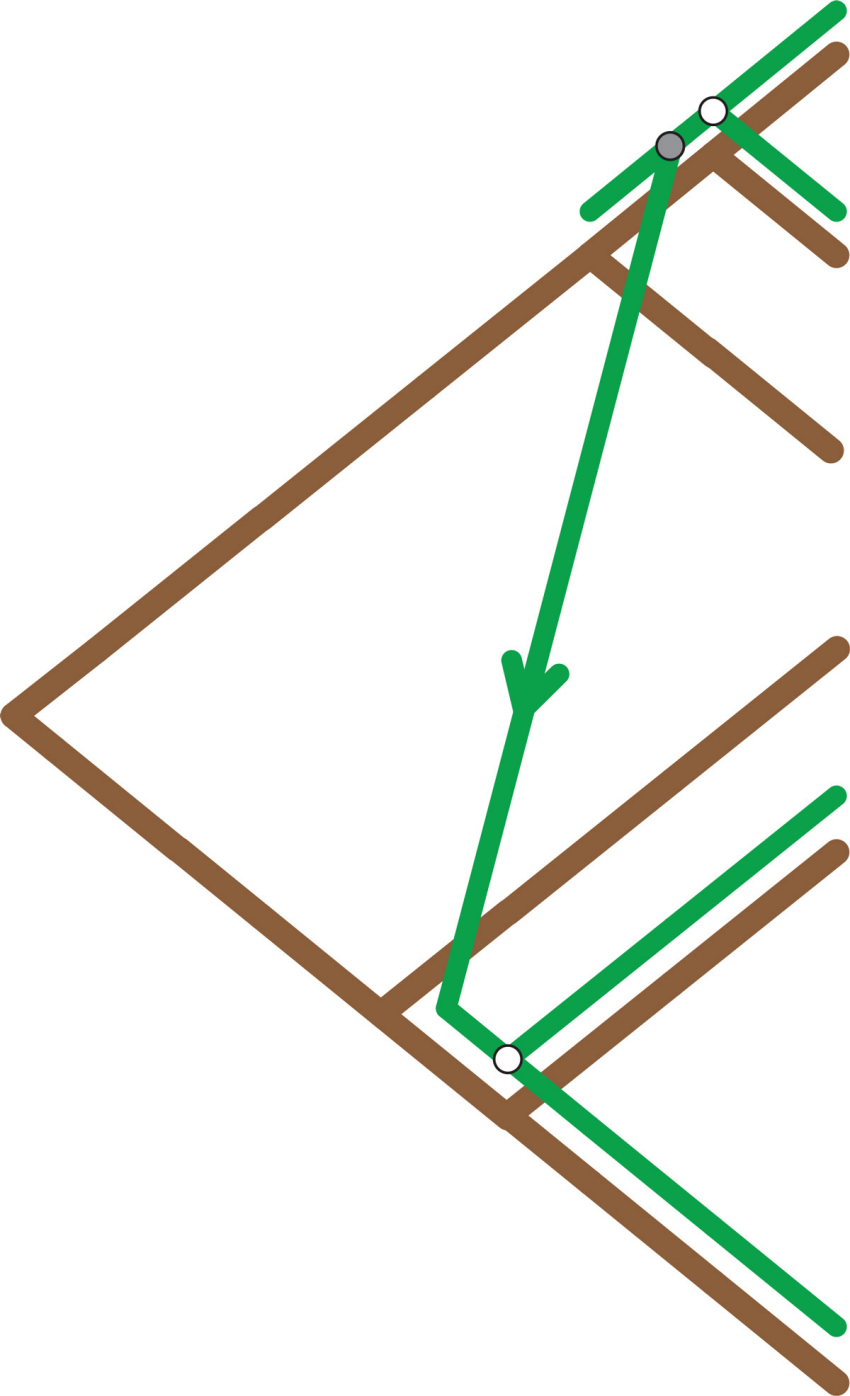
**Time Inconsistent Mapping**. This cophylogeny mapping is biologically infeasible.

An unfortunate consequence of ignoring the relative order of the internal nodes of the host tree is that recovered mappings may be time-inconsistent [31]. A time-inconsistent solution is the case where the order of the parasite divergence events contradicts the order of the internal nodes in the parasite's phylogeny and as a result the coevolutionary history of the parasite contradicts its phylogenetic history. A mapping that is time-inconsistent is biologically infeasible [32]. Such a mapping can be seen in Figure 2.

To overcome the limitation of potentially reporting time-inconsistent solutions Libeskind-Hadas and Charleston [14] proposed an algorithm where the relative ordering of the host phylogeny's internal nodes is fixed and each internal node is assigned a unique distance from the root. Under these conditions the cophylogeny reconstruction problem can be solved in polynomial time and the solutions recovered are guaranteed to be biologically feasible. The initial algorithm proposed required *O*(*n*^7^) running time [32], which significantly limited its applicability as it was unable to converge on good solutions for large problem instances. Subsequent solutions significantly reduced this running time with Edge Mapping [30], Improved Node Mapping [33], and Slicing [31,34], all running in *O*(*n*^3^).

Although polynomial time algorithms exist for solving the case where the internal nodes are fixed, the number of internal node orderings increases exponentially for every internal node added to a bifurcating tree. Therefore, such algorithms rely on a metaheuristic to iterate through the exponential number of internal node orderings. This approach has been shown to be successful, with software tools such as Jane [32] converging on solutions for coevolutionary systems with up to 200 taxa [35].

Cruaud *et al's *analysis [35] demonstrated the value of coevolutionary analysis of larger phylogenetic histories and also highlighted the need for further research of large scale coevolutionary analysis. The use of algorithms such as those implemented in Jane, however, are not feasible in all cases. Consider the coevolving system of Wolbachia and their insect hosts. It is currently estimated that 20% of all insect species are inhabited by Wolbachia [36], and that the estimated number of insects is approximately 6 million [37]. Such a problem instance represents a 6000 times increase in the number of taxa compared to the largest system successfully analysed using Jane. This is a significant increase in the complexity of the problem, especially when considering the exponential increase in complexity for every additional node added. This example demonstrates the need to develop further algorithms for the cophylogeny reconstruction problem, which while recovering biologically feasible reconstructions, are able to do so in sub *O*(*n*^3^) time.

This paper introduces a new greedy approach for solving the cophylogeny reconstruction problem where the internal node ordering is fixed. This algorithm is able to guarantee that all reported solutions are biologically feasible. Further, this approach is tested on a combination of synthetic and real data sets and is shown to recover comparable solutions to existing *O*(*n*^3^) algorithms but with a significantly reduced running time of *O*(*n*).

## Methods

The algorithm proposed incorporates a number of techniques which have been successful in their own right for solving the cophylogeny reconstruction problem and utilises them in a novel manner to tackle the problem of recovering biologically feasible reconstructions for large coevolutionary systems. The algorithm referred to here as TreeCollapse applies a combination of both pattern and event-based reconstruction techniques, with the aim of producing a fast and scalable method for solving the cophylogeny reconstruction problem for the case where the internal node order is fixed.

This algorithm is then applied to a metaheuristuc framework similar to the dynamic programming algorithm leveraged by Jane [32]. This research does not focus on improving the search strategies applied by the metaheurstic for traversing the exponential search space, but rather aims to minimise the time spent evaluating each instance. Unlike the dynamic programming algorithm used by Jane 2 and subsequent releases which requires an *O*(*n*^3^) [30] evaluation step for each instance processed by the metaheuristic, TreeCollapse recovers a cophylogeny mapping in *O*(*n*). This allows TreeCollapse to evaluate *O*(*n*^2^) instances for each case evaluated by Jane.

TreeCollapse is an extension of the pattern detection methodology first proposed by Ronquist [16]. A significant change to Ronquist's pattern detection framework is the leveraging of local pattern detection rather than Ronquist's history classification technique [16,38]. The proposed pattern detection framework bounds the search space of each pattern to a constant size by considering only a constant number of cherries at each step. A cherry (*C*) is defined by McKenzie and Steel [39] as:

**Definition 1 ***In a bifurcating tree a cherry *(*C_i_*) *is a pair of leaf nodes *(*i_left_, i_right_*) *each of which is adjacent to a common ancestor *(*i_parent_*).

The proposed framework constrains all pattern detection to both *O*(1) running time and search space, to maximise the number of unique internal host tree node orderings which can be explored in a fixed period of time. The result of the *O*(1) bound on the pattern detection is the ability of this algorithm to construct cophylogeny mappings in *O*(*n*) time and space, where all reported solutions are time-consistent; using the trade off that not all solutions recovered are from the Pareto front.

### Local pattern detection

**Figure 3:**
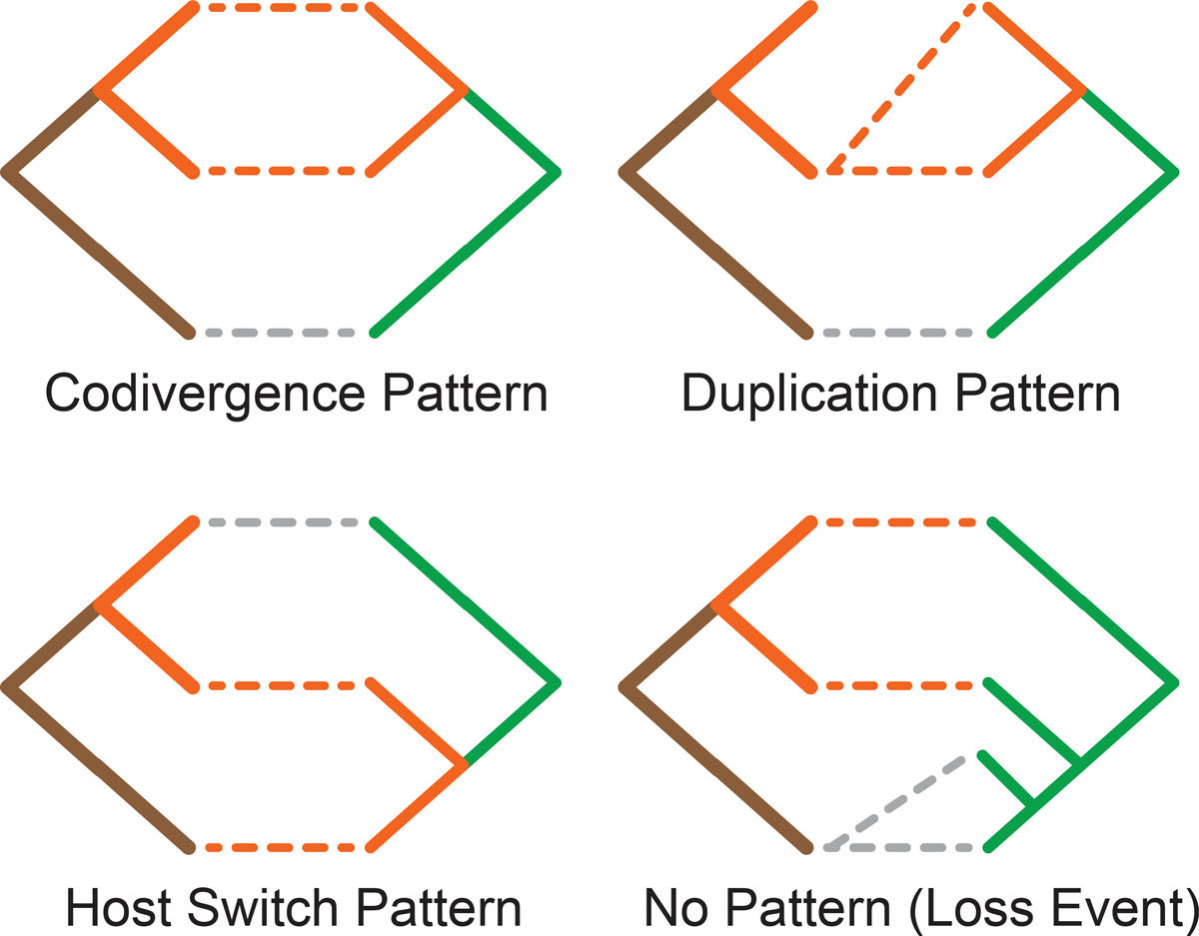
**Basic Patterns**. The basic four patterns which can be detected by TreeCollapse and their resultant mappings.

**Figure 4:**
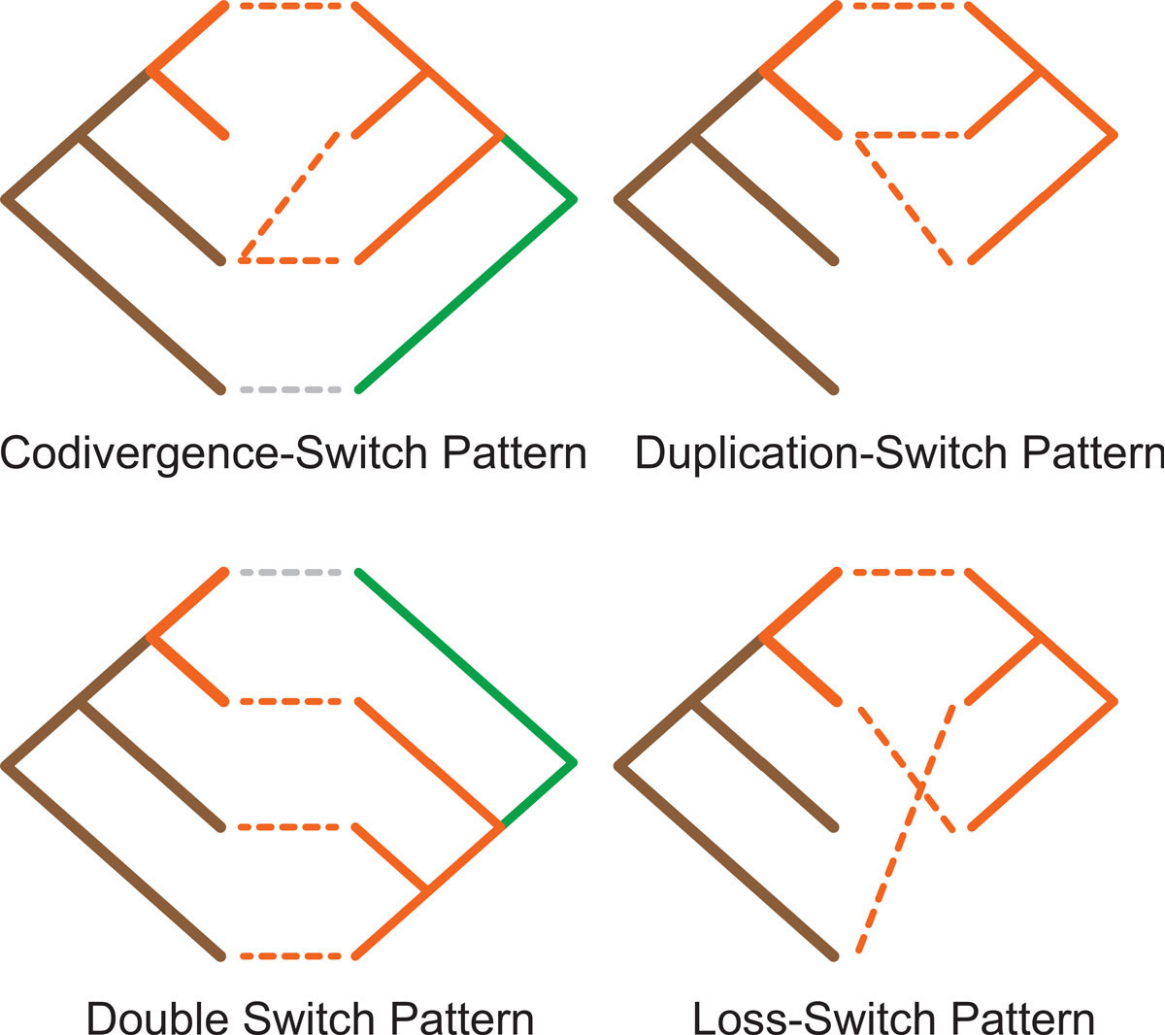
**Hybrid Patterns**. The four hybrid patterns which aim to combat the complexity of host switch events.

The proposed algorithm reconstructs a cophylogeny mapping based on patterns formed by cherries in both the host and parasite trees. The eight patterns used consist of four *base *patterns, as can be seen in Figure 3, which are used to recover the four recoverable events that can occur in a coevolutionary system. These patterns are derived from the history classification framework proposed by Ronquist [16]. An additional four patterns have been included to improve this framework's performance at handling the complexity of host switch events. These *hybrid *patterns extend the initial base patterns to allow each hybrid pattern to detect two consecutive events. The hybrid pattern set consists of *codivergence-switch, duplication-switch, loss-switch *and *double switch*, all of which can be seen in Figure 4. By including these hybrid patterns this approach is able to more accurately recover host switch events, which results in a reduction in the total parsimony score. This is achieved by only considering an additional two cherries which is clearly still bounded by *O*(1). A further in-depth analysis on the accuracy improvements achieved by the proposed hybrid patterns is discussed as part of the Results.

The patterns are detected for each cherry in the host tree by executing a depth first search. This algorithm is designed to traverse the tanglegram instance (*H, P, φ*), starting at the leaves of the host cherry and terminating once a path is found back to a leaf in the host tree. To ensure that this algorithm runs in *O*(1), the number of levels which the depth first search algorithm may traverse through the parasite tree is fixed to a constant factor. Once the algorithm has terminated, the resultant path found by the depth first search algorithm is compared to the permitted pattern set. This search algorithm is bound to *O*(1) as there is a constant number of levels searched and the branching rate of a bifurcating tree is also constant. It is important to note that this is only one approach to implementing this pattern detection algorithm in constant time.

Each pattern recovered by the local pattern detection algorithm gives rise to one or more cherries from the host or parasite trees to collapse, hence the algorithm's name TreeCollapse. This operation is based on a bottom up iteration technique, where the cherries in the host tree are sequentially processed based on their distance from the root. As a result, this algorithm continues until both trees consist of only a single node (the root of both the host and parasite tree).

The TreeCollapse local pattern detection framework allows for three different collapse functions. The first case is where both a host and parasite cherry are collapsed, which indicates a codivergence event. The second case is where a cherry from the parasite tree is collapsed, indicating either a duplication or host switch event. Finally, if no pattern is formed with the parasite tree, then a loss event is inferred and the host cherry is collapsed.

There are three possible final states when this process is performed to completion. First, the host tree and parasite tree are both collapsed up to their respective root nodes. In this case there is a codivergence event at the root of the host tree. The second case is where the host tree still has more than one node remaining after the parasite tree is completely collapsed. In this case we can ignore all remaining nodes in the host tree. Finally, the case where the host tree is completely collapsed before the parasite tree results in the remaining nodes in the parasite tree being appended before the root of the host tree as duplication events. As TreeCollapse handles all three scenarios, it is capable of recovering solutions for all instances of the cophylogeny reconstruction problem.

### Guarantee that TreeCollapse recovers time-consistent solutions

TreeCollapse requires that each cherry in the host tree be processed in decreasing order based on the node's depth. Depth is defined in this context as the distance to the root as the sum of the branch lengths. We will refer to the depth of a node *a *as *d*(*a*). To prevent timing inconsistencies, TreeCollapse requires that the edge weights in the host tree are set in such a way that each internal node's depth is unique and bound between 0 and (*n − *2), where the total number of the internal and leaf nodes in the tree is (2*n − *1). This approach has been previously applied by dynamic programming approaches to ensure that recovered solutions are biologically feasible [32,33]. The *n *leaf nodes are then assigned to a common depth of (*n − *1). Under such a construction we claim that:

**Theorem 1 ***The TreeCollapse local pattern detection and collapse framework ensures that no host switch event gives rise to time-inconsistent solutions*.

**Figure 5:**
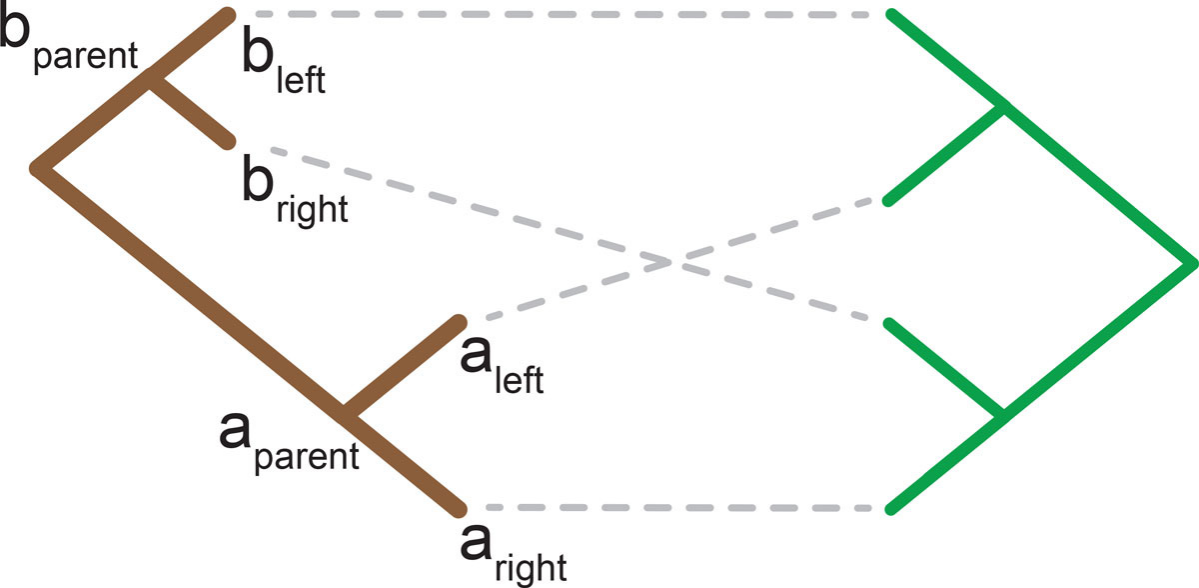
**State which induces time-inconsistent solutions**. The state where the leaves of cherry *b *are closer to the root than the parent of cherry *a*.

*Proof *By contradiction we show that all cophylogeny mappings reported by TreeCollapse are time consistent. Let us assume TreeCollapse returns cophylogeny mappings that are time-inconsistent. For this to occur requires that there exists a cherry in the host tree *C_a _*which forms a switch pattern with another node in the host tree *b *where *d*(*b*) *< d*(*a_parent_*) as seen in Figure 5.

There are two cases to consider, where *b *is an internal node in the initial host tree and where *b *is a leaf in the initial host tree. The case that *b *is an internal node in the host tree requires the cherry for which *b *was previously a parent (*C_b_*) to be collapsed before *C_a_*. This violates the condition that TreeCollapse must process the cherries in decreasing order based on the distance from the root, which is clearly not true if *C_b _*is processed before *C_a_*. Alternatively, where *b *is a leaf node from the initial host tree is also invalid as the initial depths of all leaf nodes must be greater than the depth of all internal nodes, which is not true if *d*(*b*) *< d*(*a_parent_*). Therefore, as *d*(*b*) ≮ *d*(*a_parent_*) for all valid constructions of the host, we have proved Theorem 1.

This result can be extended to all hybrid patterns as they are each derived from the switch pattern and in all cases require that the cherry whose parent has the maximum depth is the next selected. Enforcing this restriction, however, results in an approach that potentially over-counts the number of loss events. In the next section we introduce an *O*(*n*) post processing algorithm that aims to counterbalance this result by minimising the number of loss events in the final reconstruction.

### Increasing accuracy further

While the TreeCollapse algorithm guarantees that all histories reported are time-consistent, it does not give such a guarantee that reported solutions have a minimum cost, as no algorithm to date has been able to achieve this in sub *O*(*n*^3^) time.

To further minimise the cost of the resultant solution, we propose a postprocessing algorithm called Right Push which follows after the TreeCollapse algorithm. This post-processing step aims to find the optimum switch placement for a cophylogeny mapping for a specific event order where the node ordering is also fixed. This is achieved by minimising the number of loss events without introducing time-inconsistencies. The aim of this post-processing step is to reduce the number of overcounted loss events which result from ensuring that time-inconsistent switch events do not arise during the pattern detection and collapse phase.

**Figure 6:**
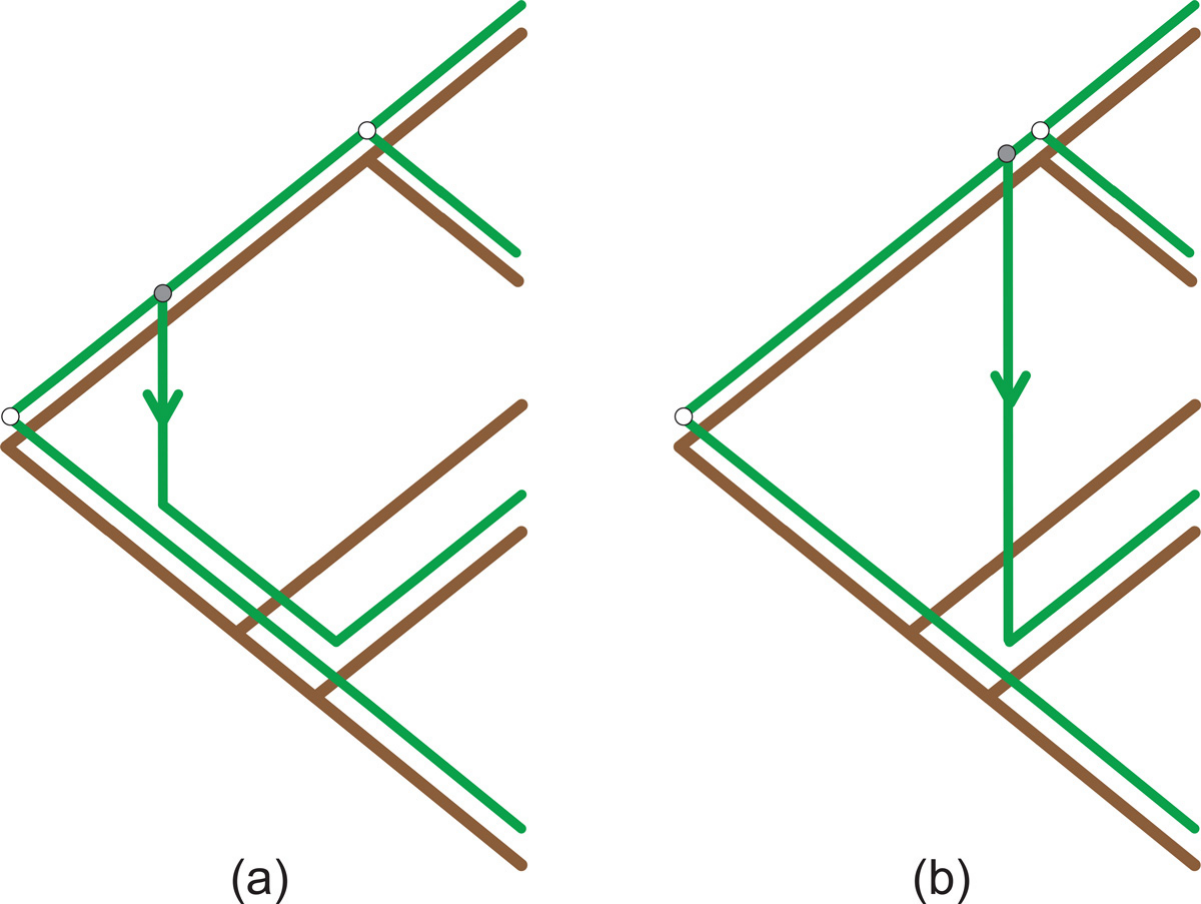
**Right Push**. An example of a cophylogeny mapping before and after right push is applied.

Right Push is a greedy algorithm which runs bottom up through the cophylogeny mapping (Φ(*P*)), shifting the host switch take-off and landing edges to the optimum position. The algorithm was named Right Push as traditionally host trees are drawn with their root on the left and their leaves on the right, as seen in Figure 1, and therefore this algorithm aims to pull host switch events towards the leaves, that is, towards the "Right". A case where Right Push minimises the number of loss events can be seen in Figure 6. This algorithm selects the optimal take-off and landing edges, while ensuring the resultant solution is time-consistent by maintaining the the properties set out in Definition 2.

**Definition 2 ***Suppose p *∈ Φ(*P*) *has children p_left _and p_right_, with *Φ(*p_left_*) = *s and *Φ(*p_right_*) = *t. Then the optimal host switch location is a pair of edges e_i _and e_j _*∈ *E*(*H*) *where the total number of loss events between *(*e_i_, s*) *and between *(*e_j _, t*) *is minimal, and where e_i _and e_j _lie in the same time interval*.

This definition, based on prior work by Drinkwater and Charleston [33], allows for the optimal take-off and landing edge for the host switch event to be recovered using the level ancestor problem [40]. Consider the children *s *and *t *where *d*(*s*) *< d*(*t*). Under this definition, we use *d*(*s*) as the search candidate using the level ancestor query function to find both *e_i _*and *e_j_*, which allows for the optimal host switch event to be recovered in *O*(1) [33].

The Right Push algorithm is therefore designed to iterate through a cophylogeny mapping (Φ(*P*)) consisting of *O*(*n*) parasite nodes. For each parasite node mapped into the host corresponding to a host switch event, the take-off and landing sites are evaluated using the Level Ancestor Problem to identify if a lower cost switch location exists. This evaluation check along with the host switch repositioning both run in constant time [33].

By shifting the host switch to the optimal position within the current cophylogeny mapping the Right Push algorithm minimises the number of loss events while maintaining the order of the current set of codivergence, duplication and host switch events, which in turn ensures that the current mapping remains time-consistent. Analysis of Right Push's impact on the accuracy of the TreeCollapse framework is discussed in detail as part of the Results.

### Complexity analysis of TreeCollapse

The previous sections describe two algorithms which have been applied together to provide a novel greedy algorithm to solve the cophylogeny reconstruction problem. In this section we prove that the running time complexity of both these algorithms is in fact *O*(*n*). To assist with the complexity analysis, we define the number of nodes in the host tree to be *O*(*m*) and the number of nodes in the parasite tree to be *O*(*k*), with the total number of nodes in both trees being *O*(*n*), which is consistent with existing complexity analyses in this field [14,15,31-34].

To compute the complexity of TreeCollapse's local pattern detection algorithm, we will break the process into three stages: firstly, setting and storing the order of cherries to process, secondly, processing each cherry, and finally, the iteration over all current and future cherries in the host tree.

The first step in the TreeCollapse algorithm requires the generation of a list of current and future cherries in the host tree, which are sorted in descending order based on their distance from the root. Generally sorting such an unordered list requires a running time of *O*(*m *log *m*). In this case, however, we can use certain properties of the fixed node orderings to show the following:

**Lemma 1 ***A list of current and future cherries in the order they will be processed can be constructed in O*(*m*), *where the number of nodes in the host tree is O*(*m*).

*Proof *The input to this algorithm is a host tree where each of the internal nodes have a set of unique indices (0,. . ., *m − *2). Therefore, this step reduces to creating a list of current and future cherries. Our first step is to recursively set the distance to the root for each node which is well established to take *O*(*m*). Using this result we can then construct an ordered list using a bucket sort where the number of buckets required is bounded by the number of unique depths in the host tree, *O*(*m*). Therefore, as the number of the buckets is bounded by *O*(*m*) the running time of the bucket sort also runs in *O*(*m*) [41].

The second stage requires the uncovering of all patterns for each cherry *C_i_*, of which there are up to *O*(*k*), as there is a pattern for each node in the parasite tree. We consider two possible approaches to process these patterns. The first, a brute force approach, is to uncover all possible patterns for each event processed. This method requires *O*(*k*^2^) time to process all the events for the cherry *C_i_*. An alternative approach is that for each cherry *C_i_*, all patterns recovered are stored as a list of patterns to be processed. If *C_i _*is collapsed before this list of patterns is processed then this list is allocated to its parent for subsequent processing. This approach, unlike the previous brute force method, requires that each pattern only be uncovered once for each node, and that the list of *O*(*k*) patterns is sequentially processed. If the second approach is applied we can prove the following:

**Lemma 2 ***The number of patterns formed at each node in the host tree is $O\left(\frac{k}{m}\right)$*

*Proof *By only recovering each pattern once the running time to detect all possible patterns for each node is reduced to *O*(*k*) to process each cherry *C_i_*. This running time can in fact be reduced further to $O\left(\frac{k}{m}\right)$ as the average running time for each internal node. This can be verified by considering the number of nodes in the parasite tree. Each pattern formed by a parasite tree cherry requires at least three nodes. Of these three nodes only one of these will be reused once the cherry is collapsed. As each cherry can only apply one pattern, then the total number of patterns which can be formed by the parasite tree is *O*(*k*). Therefore, the average number of patterns formed for each internal node on average is $O\left(\frac{k}{m}\right)$, even if it is possible for a single cherry in the host tree to have up to *O*(*k*) patterns.

Lemma 1 and Lemma 2 give rise to the following result:

**Lemma 3 ***The running time of the TreeCollapse Algorithm is O*(*k *+ *m*)

Lemma 2 establishes the running time required to process each cherry *C_i_* in the host tree. From Lemma 2 the time required to construct a cophylogeny mapping of the initial problem instance can be derived as $O\left(m\times \frac{k}{m}+m\right)=O\left(k+m\right)$, where *k *is the running time required to map the parasite tree into the host tree and *m *is the time taken to collapse any sections of the host that do not share a common coevolutionary history with the parasite tree.

The running time of TreeCollapse is also dependent on the running time of any post processing algorithms. As a result the running time of Right Push is required to establish the overall computational complexity of the TreeCollapse algorithm. We claim that the running time of Right Push is as follows:

**Lemma 4 ***The running time of the Right Push algorithm is O*(*k *+ *m*).

*Proof *The Right Push algorithm is designed to iterate over the cophylogeny mapping (Φ(*P*)). This requires iterating over a list of size *O*(*k*). At each step the mapping instance is evaluated using the query function of the level ancestor problem. The result of this query is used to update the mapping, minimising the number of loss events in the current reconstruction ordering. The running time of a query made to the level ancestor problem is known to run in *O*(1), when pre-processing has been applied to the query tree [40]; in this context, the host tree and updating the new location is an *O*(1) assignment operation.

Therefore, the Right Push algorithm runs in *O*(*pre*-*processing*) + *O*(*k*). The preprocessing step for Right Push is the complexity of the pre-processing step of the level ancestor problem. This has previously been shown to be bounded by the size of the query tree [40], which in this context is the size of the host tree *O*(*m*).

Therefore, based on Lemma 3 and 4 the running time of both TreeCollapse and Right Push is bounded by *O*(*k *+ *m*). As *n *= *k *+ *m *we immediately get:

**Theorem 2 ***The running time of the TreeCollapse's Local Pattern Detection framework and the post-processing algorithm Right Push is bounded by O*(*n*).

**Corollary 1 ***Therefore, as the space complexity of any algorithm is bounded by its time complexity, the space complexity of TreeCollapse's Local Pattern Detection framework and the post-processing algorithm Right Push is also bounded by O*(*n*).

### Applying an approximation algorithm

TreeCollapse and Right Push are algorithms designed to solve the special case of the cophylogeny reconstruction problem where the internal node order in the host tree is fixed. Therefore, TreeCollapse, similar to previous algorithms leveraging this technique, is embedded in a metaheuristic framework as a means to recover the minimum cost reconstruction.

We apply a genetic algorithm to iterate over the exponential number of node orderings. This approach was selected over other metaheuristics, such as particle swarm optimisation [42] or ant colony optimisation [43], due to genetic algorithms' proven success for this particular problem [32].

The chromosomes of the genetic algorithm represent each internal node within the host tree, with the exception of the root which always has a depth of 0 (the distance to itself). The depth range [*i, j*] allocated for each internal node is bounded by the number of ancestor nodes between the current node and the root (*i*), and the total number of internal nodes minus the number of descendants between the current node and its leaves (*j*). Therefore, *i *and *j *are constant values for all instances processed by the genetic algorithm.

Following the allocation of depths for these chromosomes, an *O*(*n*) validation step defined by Conow *et al*. [32], is run to ensure that the numerical ordering does not violate the topological ordering. This ensures that all internal nodes are assigned unique node depths between 0 and (*n − *2), and that all leaves are assigned a timing of (*n − *1).

The genetic algorithm's fitness function promotes minimum cost reconstructions based on the Jungle event cost scheme [16]. Under this cost scheme codivergence events are assigned a cost of 0, while duplication and loss events are assigned a cost of 1, and host switch events are assigned a cost of 2, representing that a host switch is made up of a duplication followed by a switch. This fitness function was selected as it best represents the default cost functions of existing tools such as CoRe-PA [29], Jane [32], and Improved Node Mapping [33]. We note, however, that any cost scheme may be used.

## Results and discussion

We evaluate the TreeCollapse algorithm in three stages. The first stage evaluates the improvements offered by the newly proposed hybrid patterns compared to the four base patterns derived from Ronquist's previous work along with the improvement in accuracy provided by the post-processing algorithm Right Push. The second stage aims to establish a baseline measurement of TreeCollapse's performance by comparing its accuracy with two polynomial approximation algorithms for this problem. In the third stage TreeCollapse is compared to well established dynamic programming algorithms for approximating the cophylogeny reconstruction problem to identify the accuracy trade off required to produce a linear time algorithm for the fixed node ordering permutation of the cophylogeny reconstruction problem. In each of these stages a combination of synthetic and real data sets are used to evaluate TreeCollapse's performance.

The synthetic data applied for this analysis was constructed under a Yule process [44] for a previous study using CoRe-Gen (Cophylogeny Generation Model) [45]. Of the 1000 synthetic coevolutionary systems, 47 were removed as they included at least one tree with only a single node, which cannot be processed correctly by some of the software tools included in this analysis, e.g. Jane. As a result, the synthetic data applied in this study consisted of 953 instances.

The real data sets used for this analysis consist of 102 previously published data sets. These test cases cover the full spectrum of coevolutionary instances including pathogens and their hosts [46], mutualistic coevolution [47], plant-insect interactions [48], mimicry between species [49], plant-fungi relationships [50], biogeography [51], and host-parasite systems [52]. Further information on each of these data sets is provided in Additional File 1 (Table S1).

This evaluation compared cophylogeny mappings based on their resultant parsimony score. This score was derived using the Jungle cost scheme [16], consistent with previous algorithmic evaluations in this field [27,32,33]. For the pattern-based methods, this required a post processing step to evaluate the total cost. For event-based methods the Jungle cost scheme was applied to the algorithm itself.

As a further evaluation step the topology of TreeCollapse's reported mappings were compared with the results of the existing algorithms to identify the similarity of the mappings produced by each method. This evaluation acts as a further benchmark to evaluate how closely the newly proposed algorithm converges on the mappings recovered by these well-established algorithms. This includes an in-depth analysis of a coevolutionary system which is of particular importance to human health, i.e. primate-malaria [6]; where CoRe-PA, Jane and TreeCollapse each recover a unique topological mapping.

### Evaluating TreeCollapse's patterns

This research builds on Ronquist's original hypothesis, that pattern-based algorithms provide an effective approximation for the cophylogeny reconstruction problem. To evaluate each pattern's additional contribution, TreeCollapse was run where the pattern detection framework consisted of the initial four base patterns plus a single additional hybrid pattern, with the aim of identifying the benefit provided by each new pattern in isolation.

The results for this analysis over both the synthetic and real data sets can be seen in Table 1. The synthetic data highlights that all additional patterns in isolation decrease the total parsimony score and that the four hybrid patterns combined provide a significant decrease to the overall parsimony score of 7.8% on average. Of note, however, is that the individual contribution is not directly proportional to the reduction achieved when they are all applied. The results for the real data set show a similar trend with the exception of the duplication-switch pattern, which indicates a slight increase in the total parsimony score. While this increase exists, the parsimony score when all four hybrid patterns are combined is reduced 11.7% on average, even further than the results achieved over the synthetic data set.

**Table 1:** The improvement offered by the four hybrid patterns shown in incremental stages.

Patterns Applied	Total Parsimony Score (Synthetic Data Sets)	Total Parsimony Score (Real Data Sets)
Basic Four	15658	1783
Basic Four + Codivergence Switch	14800	1671
Basic Four + Duplication Switch	15564	1815
Basic Four + Loss Switch	15275	1740
Basic Four + Double Switch	15440	1610
Basic Four + Hybrid Four	14443	1574

Based on the prior result and the inconsistent results surrounding the duplication-switch pattern, a more in-depth analysis was undertaken. This led to an individual analysis of each coevolutionary instance to identify whether any benefits were provided by the duplication-switch pattern to test whether if it should be removed from the TreeCollapse framework. To achieve this result each real data set instance was evaluated in terms of their distance to the Pareto front. It was confirmed that while removing duplication-switch improves the total resultant parsimony score (particularly of outliers), the number of solutions that lie on the Pareto front was reduced from 69 out of 102 (67.6%) to 67 of 102 (65.7%). As recovering Pareto optimal solutions is key, the duplication-switch pattern was retained as a default pattern within the pattern detection framework. This result, however, highlights that further work can be undertaken in both detecting other effective patterns to apply in this framework and also that pattern classification based on the particular coevolutionary instance may provide an even more robust method.

Overall, this analysis demonstrates a significant decrease in the parsimony score recovered by TreeCollapse compared to those only using the base patterns. When the results of both the synthetic and real data sets are averaged such that each group is weighted equally, it can be seen that the four hybrid patterns provide a decrease in the parsimony score of 9.7%. This is a significant result considering this is achieved without any increase in the computational complexity of the algorithm.

### Improvements provided by Right Push

To evaluate the impact of Right Push, TreeCollapse was run with and without Right Push enabled. We show that across both data sets Right Push offers an average decrease to the parsimony score of 6%, without any additional increase in the computational complexity.

When comparing the total parsimony scores for synthetic and real data sets (Table 2), it can be seen that there is a 6.6% and 5.4% decrease in the total parsimony score respectively when Right Push is enabled. This result provides strong evidence of Right Push's ability to minimise the total parsimony score. This result also demonstrates the potential that the Right Push algorithm may offer to other greedy algorithms which apply pattern-based methods to recover cophylogeny mappings. Further, while the reduction of 9.7% using patterns was achieved using all four possible events, the 6% improvement offered by Right Push is achieved by only reducing loss events.

**Table 2:** Accuracy improvement provided when the Right Push post-processing algorithm is enabled in the TreeCollapse pattern detection framework.

	Total Parsimony Score (Synthetic Data Sets)	Total Parsimony Score (Real Data Sets)
Right Push disabled (no hybrid patterns)	16006	1876
Right Push disabled	15460	1664
Right Push enabled	14443	1574

The evaluation is run on both Synthetic and Real Data Sets.

Table 2 also provides the total improvement offered by combining both the hybrid patterns and the Right Push algorithms. Over the synthetic data set it can be seen that by applying both the hybrid patterns and Right Push a decrease in the parsimony score of 9.8% is achieved. This trend continues when evaluating the performance of both the hybrid patterns and Right Push over the real data set which show a decrease of 16.1%. As a result, by applying both the hybrid patterns and the post processing algorithm Right Push, proposed in this paper an additional 12.9% decrease in the parsimony score is gained compared to the original framework proposed by Ronquist. This result is particularly significant considering that it is achieved without any increase in the computational complexity.

### Establishing a baseline

Before comparing TreeCollapse against the current methods for cophylogenetic reconstruction, we compare TreeCollapse to two polynomial time approximation algorithms which provide a baseline for TreeCollapse's performance. These algorithms include Page's Reconciled Tree Analysis and Edge Only Mapping.

Page's Reconciled Tree Analysis recovers a Pareto optimal solution which can also be found by TreeCollapse in the case where host switch patterns are ignored. Page's Reconciled Tree Analysis recovers this mapping in linear time as the order of internal nodes does not impact on the final solution as host switch events are not considered.

The second method allow for only host switch and duplication events (Edge Only Mapping) and is able to achieve a cubic running time by avoiding the computational complexity of internal node orderings. This is achieved by reconstructing a cophylogeny mapping bounded by the final timing interval (the edge set adjacent to the leaf nodes) of the host tree. A mapping bounded within this edge set can be recovered using an existing fixed node ordering algorithm such as Improved Node Mapping [33] where the values for codivergence and loss are set to infinity and any random selected fixed node ordering is applied. This method is of interest as a dynamic programming algorithm that allows for all four recoverable events will never reconstruct a cophylogeny mapping that is more expensive than the mapping recovered by this method, and, therefore, it provides an excellent baseline for new algorithms, particularly greedy algorithms that may not offer any accuracy guarantees.

These two methods both recover Pareto optimal solutions, which are generally considered excessively expensive when applying cost schemes that assign approximately the same cost to each event, such as the Jungle cost scheme [16]. Although known to often report solutions with a high global cost, these algorithms do offer a strong preliminary baseline for the performance of TreeCollapse, which is designed to recover solutions which better approximate this problem using all four recoverable events.

The results for this analysis can be seen in Table 3. These results show that for both synthetic and real data sets, TreeCollapse's performance is significantly better than both algorithms as the aim here is to minimise the total parsimony cost. If we consider the distance from the best solution recovered i.e. the assumed Pareto Front then it can be seen that over the synthetic data set, TreeCollapse is 11% more expensive compared to 31% additional expense for Page's Reconciled Tree Analysis and 50% for Edge Only Mapping. This is compared to the real data set where TreeCollapse is 6% more expensive compared to 54% for Page's Reconciled Tree Analysis and 45% for Edge Only Mapping. As an average, this shows that TreeCollapse is 8% more expensive than the optimal compared to 42% and 48% for these two baseline algorithms clearly demonstrating that TreeCollapse outperforms both these baseline algorithms.

**Table 3:** Performance of the TreeCollapse Pattern Detection Framework compared to Edge Only Mapping and Page's Reconciled Tree Analysis on both Synthetic and Real Data sets

Algorithm	Total Parsimony Score (Synthetic Data Sets)	Total Parsimony Score (Real Data Sets)
Best known score (Reported by Jane)	12869	1481
TreeCollapse	14443	1574
Page's Reconciled Tree Analysis	18603	3198
Edge Only Mapping	25825	2686

An interesting result from this analysis, that does not directly relate to this study but does offer an avenue for further research, is the contrasting results of Page's Reconciled Tree Analysis and Edge Only Mapping over the real and synthetic data sets. These results, along with the contrasting results as part of the Right Push algorithm analysis and the analysis of the duplication-switch pattern, suggest that the CoRe-Gen may not be successfully modelling existing biological coevolutionary systems, compared to the 102 real data sets used in this analysis.

### Comparing against dynamic programming algorithms

Finally, we compare TreeCollapse to CoRe-PA and Jane, the cutting edge approximation algorithms for solving the cophylogeny reconstruction problem. These two methods are responsible for the majority of recent coevolutionary analyses using cophylogeny mapping [53-58].

CoRe-PA was selected as it is the most recent algorithmic implementation that ignores the relative order of the internal nodes in the host tree. Although known to produce time-inconsistent solutions in the worst case, CoRe-PA was included to evaluate its overall performance compared to TreeCollapse.

Jane was selected, as the metaheuristic framework leveraged by TreeCollapse is derived from Jane's genetic algorithm [32]. It is, therefore, expected that the optimal node ordering arrangement will be converged upon by both algorithms at approximately the same rate, if the number of iterations and population size are consistent. Therefore, the genetic algorithms for both methods were configured to run for 100 iterations with a population size of 100; the default configuration for the current version of Jane.

Table 4 records the parsimony score for these three algorithms. These results show that CoRe-PA is able to recover the cheapest global parsimony score with Jane performing almost as well, on both the synthetic and real data sets. TreeCollapse performed the worst out of these three algorithms in both sets. This result was expected, as a quadratic time reduction in the computational complexity of Jane's algorithm must come with a minor reduction in the resultant accuracy, in this case, a reduction of 8%.

**Table 4:** Performance of the TreeCollapse Pattern Detection Framework compared to Jane and CoRe-PA on both Synthetic and Real Data sets

Algorithm	Total Parsimony Score (Synthetic Data Sets)	Total Parsimony Score (Real Data Sets)
TreeCollapse	14443	1574
Jane	12869	1481
CoRe-PA	12839	1473

Table 4 presents CoRe-PA as the best performing algorithm. To determine whether this was due to CoRe-PA recovering time-inconsistent solutions, it was necessary to individually review each case where Jane and CoRe-PA reported a cophylogeny mapping with a different parsimony score, which included 28 cases from the synthetic data set and 6 cases from the real data set. To determine whether a time-inconsistent host switch event existed, each mapping was analysed using the CoRe-PA reconstruction viewer. In all cases, a time-inconsistency was the cause for CoRe-PA recovering a reconstruction with a parsimony score less than the mapping recovered by Jane.

As a result, CoRe-PA generated time-inconsistent solutions in 2.9% of cases over the synthetic data and 5.9% of cases over the real data set. This demonstrates that CoRe-PA is often reasonably accurate at estimating cophylogeny reconstructions especially considering that it runs in polynomial time, though the result also indicates that the resultant mapping may be time-inconsistent.

Although Jane outperforms TreeCollapse, a further analysis was run to explore how often TreeCollapse was able to find a solution close to that recovered by Jane. The result of this analysis is presented in Table 5, where the number of instances where the parsimony score is equal to Jane is computed, along with the number of cases where the solution is within 1, 2, 3, or 4 or more away from Jane's reported solution.

**Table 5:** Examining the distance for each reported solution from Jane in terms of a parsimony score

Distance of reported solution from Jane	Synthetic Data Sets (953)	Real Data Sets (102)
Equal to Jane's solution	593	69
Score is one away from Jane's solution	122	13
Score is two away from Jane's solution	68	4
Score is three away from Jane's solution	55	9
Score is four away from Jane's solution	48	4
Score is five or more away from Jane's solution	67	3

These results demonstrate that while the total parsimony score is 8% (on average) away from the total reported by Jane, TreeCollapse is able to converge on Pareto optimal solutions in 65% of cases (68% for real data), and that 83% of solutions (84% for real data) are within a score of 2 from the Pareto front. This is a significant result as it demonstrates TreeCollapse's ability, with a high frequency, to recover solutions which are approximately equal to those recovered by Jane.

**Figure 7:**
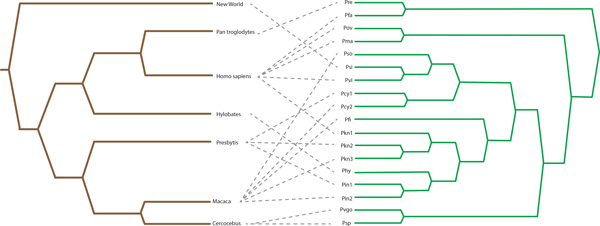
**Primate / Malaria The primate / malaria tanglegram used for this evaluation**.

It is important to note, however, that while TreeCollapse and Jane may recover mappings with an equivalent cost in 65% of cases, that the actual configuration of the mapping may not always be identical. In a number of examples, TreeCollapse and Jane reported unique mappings, which are both Pareto optimal. An example of such a case is the primate-malaria coevolutionary system (See Figure 7), which we will consider in further detail.

**Figure 8:**
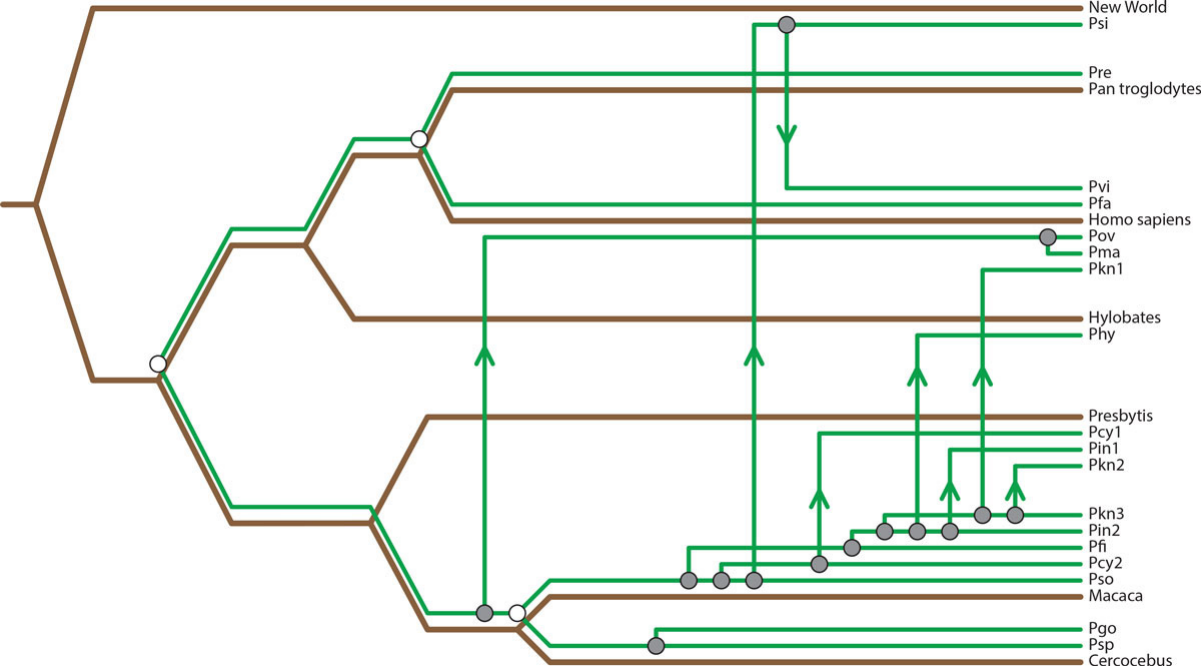
**TreeCollapse's Mapping The mapping produced by TreeCollapse for the primate / malaria tanglegram**.

**Figure 9:**
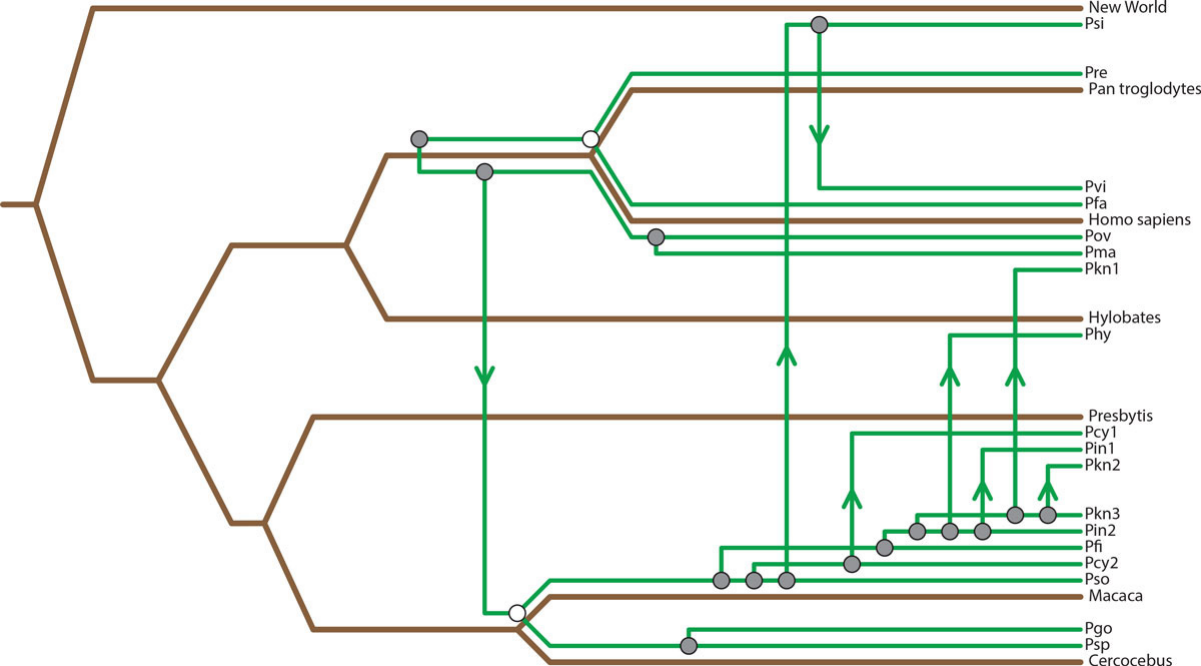
**Jane's Mapping The mapping produced by Jane for the primate / malaria tanglegram**.

**Figure 10:**
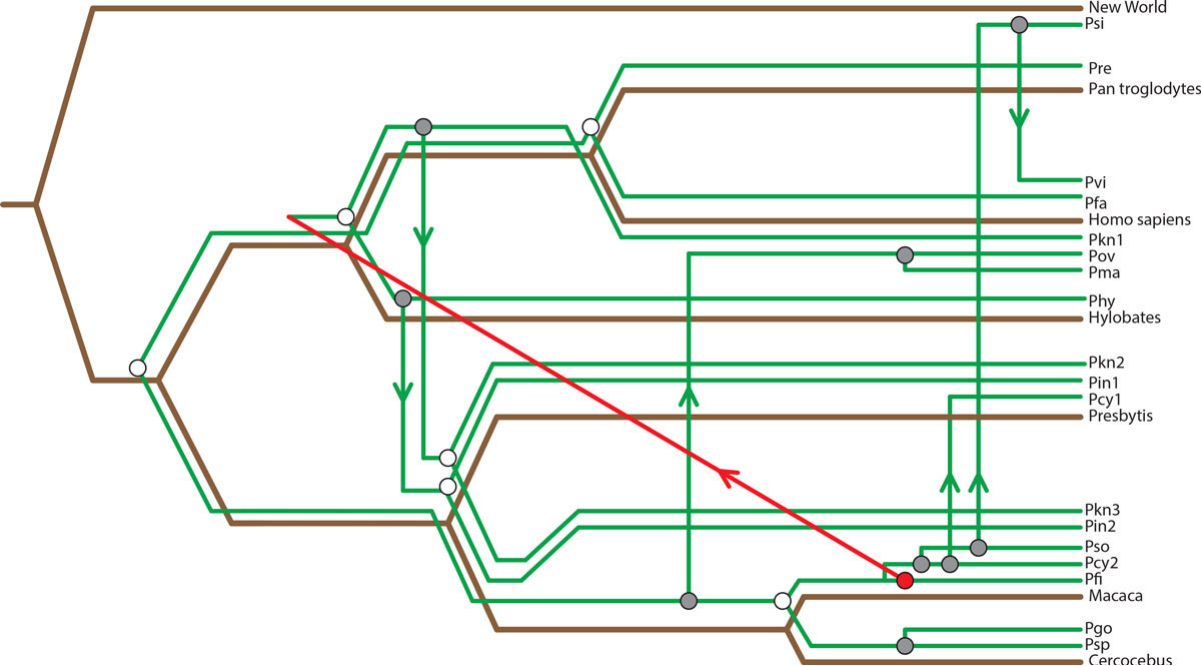
**CoRe-PA's Mapping The mapping produced by CoRe-PA for the primate / malaria tanglegram**.

The mappings recovered for the primate-malaria tanglegram for TreeCollapse, Jane and CoRe-PA can be seen in Figures 8, 9 and 10. The respective parsimony scores for each of these mappings were 24 for both TreeCollapse and Jane and 23 for CoRe-PA. Figure 10 highlights that CoRe-PA's reduced parsimony score compared to Jane and TreeCollapse is due to the time-inconsistent switch (coloured in red), which gives rise to three additional codivergence events. Jane and TreeCollpase, however, both reported solutions which are time-consistent but portray contrasting views of the origin of the parasite divergence within the host phylogeny, with TreeCollapse suggesting a longer evolutionary history between primates and malaria. This mapping further indicates a higher number of codivergence events, resulting in a more congruent reconstruction.

The unique mapping recovered for this coevolutionary system by TreeCollapse demonstrates its value as a complementary mapping algorithm for coevolutionary analyses. In this instance, by using both TreeCollapse and Jane, a larger sub-set of the Pareto front could be recovered, compared to what was achieved by using either method alone. This result affirms the value of TreeCollapse, not only as a faster way in which to approximate the cophylogeny reconstruction problem compared to approaches such as Jane, but may potentially provide further insight into coevolutionary instances.

## Conclusion

By building on the work of Ronquist, Libeskind-Hadas and Charleston, and Conow *et al*, this work presents a novel greedy algorithm for solving the cophylogeny reconstruction problem where the internal node ordering is fixed, in linear time. The reported solutions, while on average 8% more expensive than those reported by Jane, are produced by an algorithm with a quadratic running time reduction. The reported results, while produced by a linear time algorithm guarantee, all reported solutions are biologically feasible, as opposed to existing quadratic time algorithms such as CoRe-PA. Further, while Jane may recover cheaper mappings than TreeCollapse in some cases, the newly proposed framework is able to uncover optimal solutions from the Pareto front that are not reported by Jane. We therefore assert that TreeCollapse is an algorithm that complements the set of existing tools for cevolutionary analysis. Finally, we have set a baseline for algorithms which solve the fixed node ordering problem in linear time, of 67.6% accuracy over 102 biological data sets. This baseline affirms Ronquist's earlier hypothesis that pattern based reconstruction frameworks offer the potential to recover accurate approximations for host-parasite systems. Furthermore, this suggests that further work in this field using pattern based methodologies, may result in even more efficient linear time algorithms for solving cophylogeny reconstruction problem, where the internal node ordering is fixed.

## Availability and requirements

TreeCollapse, along with the real data set supplementary material, is available at http://sydney.edu.au/engineering/it/~mcharles/. TreeCollapse runs on any machine running Java 1.6 or higher.

## Competing interests

The authors declare that they have no competing interests.

## Authors' contributions

BD is responsible for the creation and implementation of the TreeCollapse algorithm and framework along with contributing to this manuscript. MC is responsible for the original problem definition and contributed to this manuscript.

## Supplementary Material

Additional file 1 **This file contains the references and brief description of the 102 real data sets used for validation of TreeCollapse**. Click here for file

## Declarations

The publication of this article was paid for by the University of Sydney.

This article has been published as part of *BMC Bioinformatics *Volume 15 Supplement 16, 2014: Thirteenth International Conference on Bioinformatics (InCoB2014): Bioinformatics. The full contents of the supplement are available online at http://www.biomedcentral.com/bmcbioinformatics/supplements/15/S16.
