# Supplementary material for: Introducing TreeCollapse: a novel greedy algorithm to solve the cophylogeny reconstruction problem
Source: BMC Bioinformatics. 2014 Dec 8;15(Suppl 16):S14. doi: 10.1186/1471-2105-15-S16-S14 (PMC4290644; doi:10.1186/1471-2105-15-S16-S14)
Supplement: Additional file 1 — This file contains the references and brief description of the 102 real data sets used for validation of TreeCollapse. [file 1471-2105-15-S16-S14-S1.pdf]

An extensive list of coevolving systems

Benjamin Drinkwater

September 17, 2014

The field of cophylogenetics has long suffered from a lack of real data sets for testing new algorithms. The largest analysis of real data sets by any algorithm has consisted of only six data sets [1, 2] and has often only considered one or two data sets when evaluating a new methodology [3, 4, 5]. This list of data sets aims to mitigate this by providing a catalogue of various tanglegrams formatted in a number of standardised way.

This data set includes 102 real data sets which represent varying degrees of coevolutionary interactions. This set includes biological phenomena consisting of host–pathogen systems, plant–insect interactions, parasitism, mimicry between species, biogeography, plant–fungi relationships and mutualistic coevolution between both species. This data set has been reconstructed from a 48 different journal and conference papers from a wide range of topics from virology through to invertebrate systematics. It is important to note that this set of Tanglegrams consists of a large sample of host–pathogen systems, with a particular focus on mammals (40%). This is due to the risk zoonoses pose to human health with 75% of emerging deaseses coming in the form of viruses which switch from other species [6].

The aim of the data set is to complement existing synthetic data sets such as those generated under a Yule process (such as those generated by CoRe-Gen [7]) such that both can be used to evaluate the success of newly proposed algorithms for coevolutionary analysis. The catalogued dataset is available for download at `http://sydney.edu.au/engineering/it/~mcharles/` in the standard nexus format (.nex), CoRe–PA Nexus format (.nex) and Tree file format (.tree).

Table S1: A list of 102 coevolving systems and their respective categories.

No.	Coevolving System	System
1	Amphibian / Polystomatidae [8]	Biogeography
2	Anolis / Plasmodium Azurophilum (Red) [9]	Host–Pathogen
3	Anolis / Plasmodium Azurophilum (White) [9]	Host–Pathogen
4	Anthophila / Nosema [10]	Parasitism
5	Aves (Seabird) / Phthiraptera [11] (A:B)	Parasitism
6	Aves (Seabird) / Phthiraptera [11] (A:C)	Parasitism
7	Aves (Seabird) / Phthiraptera [11] (A:D)	Parasitism
8	Aves (Seabird) / Phthiraptera [12]	Parasitism
9	Aves (Seabird) / Phthiraptera [12]	Parasitism
10	Aves (Seabirds) / Ischnoceran [13]	Parasitism
11	Aves (Waterbirds) / Anaticola and Aquanirmus	Parasitism
12	Aves / Avian Sarcoma Leucosis [14]	Host–Pathogen
13	Aves / Docophoroides [13]	Parasitism
14	Aves / Docophoroides [13]	Parasitism
15	Aves / Episbates, Harrisoniella, and Perineus [13]	Parasitism
16	Aves / Halipeurus [13]	Parasitism
17	Aves / Murine leukemia virus [15]	Host–Pathogen
18	Aves / Paraclisis [13]	Parasitism
19	Bemisia tabaci / Arsenophonus (23S) [16]	Parasitism
20	Bemisia tabaci / Cardinium (16S) [16]	Parasitism
21	Bemisia tabaci / P-Endosymbiont (16S) [16]	Parasitism
22	Bemisia tabaci / Wolbachia (ftsZ) [16]	Parasitism
23	Bemisia tabaci / Wolbachia (wsp) [16]	Parasitism
24	Caryophyllaceae / Microbotryum [17]	Plant–Fungi
25	Chiroptera / γretroviruses [18]	Host–Pathogen
26	Crinoidea / Myzostomida [19]	Parasitisms
27	Crinoidea / Myzostomida [19]	Parasitisms
28	Diplopoda / Acari [20]	Parasitism
29	Estrilda / Vidua [5, 21]	Mimicry
30	Ficus / Agaonidae (A) [22]	Mutualism
31	Ficus / Agaonidae (B) [22]	Mutualism
32	Ficus / Apocryptophagus [22]	Mutualism
33	Ficus / Ceratosolen [22]	Mutualism
34	Ficus / Pegoscapus [23]	Mutualism
35	Formicidae / Myrmarachne [24]	Mimicry
36	Fungus / Chrysoviridae [25]	Host–Pathogen
37	Geomyidae / Mallophaga [26]	Parasitism
38	Geomyidae / Mallophaga [26]	Parasitism
39	Geomyidae / Mallophaga [26]	Parasitism
40	Geomyidae / Mallophaga [27]	Parasitism
41	Geomyidae / Mallophaga [28]	Parasitism
42	Geomyidae / Mallophaga [4]	Parasitism

43	H. melpomene / H. erato [29]	Mimicry
44	Heterorhabditis / Photorhabdus [30]	Parasitism
45	Homo sapiens / Actinobacillus (P1:ML)[31]	Host–Pathogen
46	Homo sapiens / Actinobacillus (P1:MP)[31]	Host–Pathogen
47	Homo sapiens / Actinobacillus (P2:ML) [31]	Host–Pathogen
48	Homo sapiens / Actinobacillus (P2:MP)[31]	Host–Pathogen
49	Homo sapiens / Actinobacillus (P3:ML) [31]	Host–Pathogen
50	Homo sapiens / Actinobacillus (P3:MP) [31]	Host–Pathogen
51	Homo sapiens / JC polyomavirus [32]	Host–Pathogen
52	K. rugosus / K. waterhousei [33]	Plant–Insect
53	Magnoliophyta / RNA virus [34]	Host–Pathogen
54	Mammalia / Caliciviridae [35]	Host–Pathogen
55	Mammalia / Lyssavirus [14]	Host–Pathogen
56	Mammalia / Murine leukemia virus [15]	Host–Pathogen
57	Mammalia / Murine leukemia virus [15]	Host–Pathogen
58	Mammalia / Murine leukemia virus [15]	Host–Pathogen
59	Mammalia / Murine leukemia virus [36, 15]	Host–Pathogen
60	Mammalia / Murine leukemia virus [36]	Host–Pathogen
61	Mammalia / Narnavirdae [35]	Host–Pathogen
62	Mammalia / Partitiviridae [35]	Host–Pathogen
63	Mammalia / Polyomaviridae [37]	Host–Pathogen
64	Mammalia / Totiviridae [35]	Host–Pathogen
65	Pelecaniformes / Pectinopygus [38]	Parasitism
66	Pleistodontes / Sycoscapter [22]	Mimicry
67	Primate / Enterobius [39]	Parasitism
68	Primate / Enterobius [40]	Parasitism
69	Primate / Lentivirus [41]	Host–Pathogen
70	Primate / Oxyuroid [42]	Parasitism
71	Primate / Plasmodium (Malaria) [43]	Host–Pathogen
72	Primates / α-hepesvirus (VCV) [44]	Host–Pathogen
73	Primates / β-hepesvirus (CMV) [44]	Host–Pathogen
74	Primates / γ-hepesvirus (LCV) [44]	Host–Pathogen
75	Primates / γ-hepesvirus (RV) [44]	Host–Pathogen
76	Primates / Spumavirus [14]	Host–Pathogen
77	Primates and Bovinae / Papillomavirus [44]	Host–Pathogen
78	Rhinotermitidae / Pseudotrichonympha [45]	Mutualism
79	Rodentia / Anoplura [46]	Parasitism
80	Rodentia / Arenavirus [47]	Host–Pathogen
81	Rodentia / Arenavirus [14]	Host–Pathogen
82	Rodentia / Enterobius [48] (Restricted A)	Parasitism
83	Rodentia / Enterobius [48] (Restricted B)	Parasitism
84	Rodentia / Enterobius [48] (Restricted C)	Parasitism
85	Rodentia / Enterobius [48] (Unrestricted A)	Parasitism
86	Rodentia / Enterobius [48] (Unrestricted B)	Parasitism
87	Rodentia / Hantivirus [49]	Host–Pathogen
88	Rodentia / Hantivirus [14]	Host–Pathogen

89	Rodentia / Hantivirus [50] (Laa)	Host–Pathogen
90	Rodentia / Hantivirus [50] (Maa)	Host–Pathogen
91	Rodentia / Hantivirus [50] (Saa)	Host–Pathogen
92	Rodentia / Hantivirus [51]	Host–Pathogen
93	Rodentia / Hantivirus [52]	Host–Pathogen
94	Rodentia / Murine leukemia virus[15]	Host–Pathogen
95	Steinernematidae / Xenorhabdus [53]	Mutualism
96	Tephritinae / Symbiotic Bacteria [54]	Mutualism
97	Tephritinae / Symbiotic Bacteria [54]	Mutualism
98	Tetraspanin Uroplakins Coevolution (UP1aUPII)	Mutualism
99	Tetraspanin Uroplakins Coevolution (UP1aUPIII)	Mutualism
100	Tetraspanin Uroplakins Coevolution (UP1bUPII)	Mutualism
101	Tetraspanin Uroplakins Coevolution (UP1BUPIII)	Mutualism
102	Vertebrata / Atadenovirus [44]	Host–Pathogen

References

[1] C. Conow, D. Fielder, Y. Ovadia, and R. Libeskind-Hadas, "Jane: a new tool for the cophylogeny reconstruction problem," *Algorithms for Molecular Biology*, vol. 5, no. 1, p. 16, 2010.

[2] B. Drinkwater and M. A. Charleston, "An Improved Node Mapping Algorithm for the Cophylogeny Reconstruction Problem," *Coevolution*, vol. 2, no. 1, pp. 1–17, 2014.

[3] M. Charleston, "Jungles: A new solution to the Host/Parasite Phylogeny Reconciliation Problem," *Mathematical Biosciences*, vol. 149, no. 2, pp. 191–223, 1998.

[4] P. Legendre, Y. Desdevises, and E. Bazin, "A statistical test for host–parasite coevolution," *Systematic Biology*, vol. 51, no. 2, pp. 217–234, 2002.

[5] D. Merkle and M. Middendorf, "Reconstruction of the cophylogenetic history of related phylogenetic trees with divergence timing information," *Theory in Biosciences*, vol. 123, no. 4, pp. 277–299, 2005.

[6] L. H. Taylor, S. M. Latham, and E. Mark, "Risk factors for human disease emergence," *Philosophical Transactions of the Royal Society of London. Series B: Biological Sciences*, vol. 356, no. 1411, pp. 983–989, 2001.

[7] S. Keller-Schmidt, N. Wieseke, K. Klemm, and M. Middendorf, "Evaluation of host parasite reconciliation methods using a new approach for cophylogeny generation," tech. rep., Bioinformatics Leipzig, 2011.

[8] M. Badets, I. Whittington, F. Lalubin, J.-F. Allienne, J.-L. Maspimby, S. Bentz, L. H. Du Preez, D. Barton, H. Hasegawa, V. Tandon, *et al.*, "Correlating early evolution of parasitic platyhelminths to gondwana breakup," *Systematic biology*, vol. 60, no. 6, pp. 762–781, 2011.

[9] M. Charleston and S. Perkins, "Lizards, malaria, and jungles in the caribbean," *Tangled Trees: Phylogeny, Cospeciation and Coevolution*, pp. 65–92, 2003.

[10] A. B. Shafer, G. R. Williams, D. Shutler, R. E. Rogers, and D. T. Stewart, "Cophylogeny of nosema (microsporidia: Nosematidae) and bees (hymenoptera: Apidae) suggests both cospeciation and a host-switch," *Journal of Parasitology*, vol. 95, no. 1, pp. 198–203, 2009.

[11] A. Paterson, G. Wallis, L. Wallis, and R. Gray, "Seabird and louse coevolution: Complex histories revealed by 12s rrna sequences and reconciliation analyses," *Systematic Biology*, vol. 49, no. 3, pp. 383–399, 2000.

[12] A. Paterson and J. Banks, "Analytical approaches to measuring cospeciation of host and parasites: through a glass, darkly," *International journal for parasitology*, vol. 31, no. 9, pp. 1012–1022, 2001.

[13] R. D. Page, R. H. Cruickshank, M. Dickens, R. W. Furness, M. Kennedy, R. L. Palma, and V. S. Smith, “Phylogeny of *Philoceanus complex* seabird lice (Phthiraptera: Ischnocera) inferred from mitochondrial dna sequences,” *Molecular phylogenetics and evolution*, vol. 30, no. 3, pp. 633–652, 2004.

[14] A. Jackson and M. Charleston, “A cophylogenetic perspective of rna–virus evolution,” *Molecular biology and evolution*, vol. 21, no. 1, pp. 45–57, 2004.

[15] J. Martin, P. Kabat, and M. Tristem, “Cospeciation and horizontal transmission rates in the murine leukaemia-related retroviruses,” *Tangled trees. University of Chicago Press, Chicago*, pp. 174–194, 2002.

[16] M. Z. Ahmed, P. J. De Barro, S.-X. Ren, J. M. Greeff, and B.-L. Qiu, “Evidence for horizontal transmission of secondary endosymbionts in the bemisia tabaci cryptic species complex,” *PloS one*, vol. 8, no. 1, p. e53084, 2013.

[17] G. Refrégier, M. Le Gac, F. Jabbour, A. Widmer, J. A. Shykoff, R. Yockteng, M. E. Hood, and T. Giraud, “Cophylogeny of the anther smut fungi and their caryophyllaceous hosts: Prevalence of host shifts and importance of delimiting parasite species for inferring cospeciation,” *BMC Evolutionary Biology*, vol. 8, no. 1, p. 100, 2008.

[18] J. Cui, M. Tachedjian, L. Wang, G. Tachedjian, L. Wang, and S. Zhang, “Discovery of retroviral homologs in bats: implications for the origin of mammalian gammaretroviruses,” *Journal of virology*, vol. 86, no. 8, pp. 4288–4293, 2012.

[19] D. Lanterbecq, G. W. Rouse, and I. Eeckhaut, “Evidence for cospeciation events in the host–symbiont system involving crinoids (echinodermata) and their obligate associates, the myzostomids (myzostomida, annelida),” *Molecular phylogenetics and evolution*, vol. 54, no. 2, pp. 357–371, 2010.

[20] L. Swafford and J. E. Bond, “The symbiotic mites of some appalachian xystodesmidae (diplopoda: Polydesmida) and the complete mitochondrial genome sequence of the mite stylochyrus rarior (berlese)(acari: Mesostigmata: Ologamasidae),” *Invertebrate Systematics*, vol. 23, no. 5, pp. 445–451, 2010.

[21] M. Sorenson, C. Balakrishnan, and R. Payne, “Clade-limited colonization in brood parasitic finches (vidua spp.),” *Systematic Biology*, vol. 53, no. 1, pp. 140–153, 2004.

[22] A. Jackson, “Cophylogeny of the ficus microcosm,” *Biological Reviews*, vol. 79, no. 4, pp. 751–768, 2004.

[23] A. Jackson, C. Machado, N. Robbins, E. Herre, *et al.*, “Multi-locus phylogenetic analysis of neotropical figs does not support co-speciation with the

pollinators: the importance of systematic scale in fig/wasp cophylogenetic studies," *Symbiosis (Rehovot)*, vol. 45, no. 1, p. 57, 2008.

[24] C. B. Firth, *An Evolutionary Perspective on the Origin and Spread of Emerging Infectious Diseases.* PhD thesis, College of Science, 2010.

[25] M. Göker, C. Scheuner, H.-P. Klenk, J. B. Stielow, and W. Menzel, "Codivergence of mycoviruses with their hosts," *PloS one*, vol. 6, no. 7, p. e22252, 2011.

[26] J. Demastes, T. Spradling, and M. Hafner, "The effects of spatial and temporal scale on analyses of cophylogeny," *Tangled Trees: Phylogeny, Cospeciation, and Coevolution*, p. 221, 2002.

[27] M. S. Hafner and S. A. Nadler, "Phylogenetic trees support the coevolution of parasites and their hosts," 1988.

[28] M. Hafner, J. Demastes, T. Spradling, D. Reed, and R. Page, "Cophylogeny between pocket gophers and chewing lice," *Tangled Trees: phylogeny, cospeciation and coevolution*, pp. 195–220, 2003.

[29] J. Cuthill and M. Charleston, "Phylogenetic codivergence supports coevolution of mimetic heliconius butterflies," *PloS one*, vol. 7, no. 5, p. e36464, 2012.

[30] P. Maneesakorn, R. An, H. Daneshvar, K. Taylor, X. Bai, B. J. Adams, P. S. Grewal, and A. Chandrapatya, "Phylogenetic and cophylogenetic relationships of entomopathogenic nematodes (¡ i¿ heterorhabditis¡/i¿: Rhabditida) and their symbiotic bacteria (¡ i¿ photorhabdus¡/i¿: Enterobacteriaceae)," *Molecular phylogenetics and evolution*, vol. 59, no. 2, pp. 271–280, 2011.

[31] P. J. Planet, S. C. Kachlany, D. H. Fine, R. DeSalle, and D. H. Figurski, "The widespread colonization island of actinobacillus actinomycetemcomitans," *Nature genetics*, vol. 34, no. 2, pp. 193–198, 2003.

[32] L. A. Shackelton, A. Rambaut, O. G. Pybus, and E. C. Holmes, "Jc virus evolution and its association with human populations," *Journal of virology*, vol. 80, no. 20, pp. 9928–9933, 2006.

[33] M. McLeish, B. Crespi, T. Chapman, and M. Schwarz, "Parallel diversification of Australian gall-thrips on *Acacia*," *Molecular phylogenetics and evolution*, vol. 43, no. 3, pp. 714–725, 2007.

[34] I. Pagán, C. Firth, and E. C. Holmes, "Phylogenetic analysis reveals rapid evolutionary dynamics in the plant rna virus genus tobamovirus," *Journal of molecular evolution*, vol. 71, no. 4, pp. 298–307, 2010.

[35] G. J. Etherington, S. M. Ring, M. A. Charleston, J. Dicks, V. J. Rayward-Smith, and I. N. Roberts, "Tracing the origin and co-phylogeny of the caliciviruses," *Journal of general virology*, vol. 87, no. 5, pp. 1229–1235, 2006.

[36] R. A. Weiss, "The leeuwenhoek lecture 2001. animal origins of human infectious disease," *Philosophical Transactions of the Royal Society of London. Series B: Biological Sciences*, vol. 356, no. 1410, pp. 957–977, 2001.

[37] M. Pérez-Losada, R. G. Christensen, D. A. McClellan, B. J. Adams, R. P. Viscidi, J. C. Demma, and K. A. Crandall, "Comparing phylogenetic codivergence between polyomaviruses and their hosts," *Journal of virology*, vol. 80, no. 12, pp. 5663–5669, 2006.

[38] J. Hughes, M. Kennedy, K. P. Johnson, R. L. Palma, and R. D. Page, "Multiple cophylogenetic analyses reveal frequent cospeciation between pelecaniform birds and pectinopygus lice," *Systematic biology*, vol. 56, no. 2, pp. 232–251, 2007.

[39] J. Hugot, "Primates and their pinworm parasites: the cameron hypothesis revisited," *Systematic Biology*, vol. 48, no. 3, pp. 523–546, 1999.

[40] F. RONQUIST, "Phylogenetic approaches in coevolution and biogeography," *Zoologica scripta*, vol. 26, no. 4, pp. 313–322, 2005.

[41] M. Charleston and D. Robertson, "Preferential host switching by primate lentiviruses can account for phylogenetic similarity with the primate phylogeny," *Systematic biology*, vol. 51, no. 3, pp. 528–535, 2002.

[42] S. Morand and R. Poulin, "Phylogenies, the comparative method and parasite evolutionary ecology," *Advances in parasitology*, vol. 54, pp. 281–302, 2003.

[43] J. Mu, D. Joy, J. Duan, Y. Huang, J. Carlton, J. Walker, J. Barnwell, P. Beerli, M. Charleston, O. Pybus, *et al.*, "Host switch leads to emergence of plasmodium vivax malaria in humans," *Molecular biology and evolution*, vol. 22, no. 8, pp. 1686–1693, 2005.

[44] A. Jackson, "The effect of paralogous lineages on the application of reconciliation analysis by cophylogeny mapping," *Systematic biology*, vol. 54, no. 1, pp. 127–145, 2005.

[45] S. Noda, O. Kitade, T. Inoue, M. Kawai, M. Kanuka, K. Hiroshima, Y. Hongoh, R. Constantino, V. Uys, J. Zhong, *et al.*, "Cospeciation in the triplex symbiosis of termite gut protists (pseudotrichonympha spp.), their hosts, and their bacterial endosymbionts," *Molecular ecology*, vol. 16, no. 6, pp. 1257–1266, 2007.

[46] V. S. SMITH, J. E. LIGHT, and L. A. DURDEN, "Rodent louse diversity, phylogeny, and cospeciation in the manu biosphere reserve, peru," *Biological Journal of the Linnean Society*, vol. 95, no. 3, pp. 598–610, 2008.

[47] D. Coulibaly-N'Golo, B. Allali, S. K. Kouassi, E. Fichet-Calvet, B. Becker-Ziaja, T. Rieger, S. Ölschläger, H. Dosso, C. Denys, J. Ter Meulen, *et al.*, "Novel arenavirus sequences in hylomyscus sp. and mus (nannomys) setulosus from côte d'ivoire: Implications for evolution of arenaviruses in africa," *PloS one*, vol. 6, no. 6, p. e20893, 2011.

[48] J. Hugot, "New evidence of hystricognath rodents monophyly from the phylogeny of their pinworms," *Tangled Trees: Phylogeny, Cospeciation and Coevolution (RDM Page, ed.). The University of Chicago press, Chicago, London*, pp. 144–173, 2002.

[49] W.-P. Guo, X.-D. Lin, W. Wang, J.-H. Tian, M.-L. Cong, H.-L. Zhang, M.-R. Wang, R.-H. Zhou, J.-B. Wang, M.-H. Li, *et al.*, "Phylogeny and origins of hantaviruses harbored by bats, insectivores, and rodents," *PLoS pathogens*, vol. 9, no. 2, p. e1003159, 2013.

[50] H. J. Kang, S. N. Bennett, A. G. Hope, J. A. Cook, and R. Yanagihara, "Shared ancestry between a newfound mole-borne hantavirus and hantaviruses harbored by cricetid rodents," *Journal of virology*, vol. 85, no. 15, pp. 7496–7503, 2011.

[51] X.-D. Lin, W. Wang, W.-P. Guo, X.-H. Zhang, J.-G. Xing, S.-Z. Chen, M.-H. Li, Y. Chen, J. Xu, A. Plyusnin, *et al.*, "Cross-species transmission in the speciation of the currently known murinae-associated hantaviruses," *Journal of Virology*, vol. 86, no. 20, pp. 11171–11182, 2012.

[52] O. Vapalahti, Å. Lundkvist, V. Fedorov, C. J. Conroy, S. Hirvonen, A. Plyusnina, K. Nemirov, K. Fredga, J. A. Cook, J. Niemimaa, *et al.*, "Isolation and characterization of a hantavirus from lemmus sibiricus: evidence for host switch during hantavirus evolution," *Journal of virology*, vol. 73, no. 7, pp. 5586–5592, 1999.

[53] M.-M. Lee and S. P. Stock, "A multilocus approach to assessing coevolutionary relationships between steinernema spp.(nematoda: Steinernematidae) and their bacterial symbionts xenorhabdus spp.(γ-proteobacteria: Enterobacteriaceae)," *Systematic parasitology*, vol. 77, no. 1, pp. 1–12, 2010.

[54] L. Mazzon, I. Martinez-Sañudo, M. Simonato, A. Squartini, C. Savio, and V. Girolami, "Phylogenetic relationships between flies of the tephritinae subfamily (diptera, tephritidae) and their symbiotic bacteria," *Molecular Phylogenetics and Evolution*, vol. 56, no. 1, pp. 312–326, 2010.
